# Scaling injustice: epistemic harm in DID and what clinical records will teach AI

**DOI:** 10.3389/fpsyt.2026.1741240

**Published:** 2026-03-02

**Authors:** Oluwafunmilayo Akinlade

**Affiliations:** 1 Department of Psychiatry and Neurobehavioral Sciences, University of Virginia, Charlottesville, VA, United States; 2 Dissociative Disorders and Trauma Research Program, McLean Hospital, Belmont, MA, United States

**Keywords:** artificial intelligence in psychiatry, clinical documentation, diagnostic bias, dissociative identity disorder, electronic health records, epistemic injustice and psychiatry, trauma-informed care

## Abstract

Dissociative Identity Disorder (DID) remains one of psychiatry’s most doubted diagnoses, where patients’ accounts are dismissed and their experiences forced into ill-fitting diagnostic categories. This article examines how testimonial and hermeneutical injustices manifest in clinical practice, from skepticism about the disorder’s validity to documentation that renders patients’ trauma histories incoherent. These failures delay accurate diagnosis, erode therapeutic alliances, and create clinical records that now train artificial intelligence systems. As AI tools increasingly shape psychiatric decision-making, we face an urgent reality: if clinicians cannot recognize or document complex trauma accurately, automated systems will scale these failures exponentially. Drawing on DID research and epistemic justice frameworks, I argue for immediate reforms in clinical documentation, psychiatric training, and data governance to prevent algorithmic amplification of longstanding harms.

## Introduction

A patient receives their sixth diagnosis in eight years. The first was major depression, then bipolar II, then borderline personality disorder. Every medication trial fails. Every new clinician reads “non-adherent” and “presents inconsistently” in previous notes. What none documented: childhood trauma, or dissociative episodes mistaken for mood instability. Now, this patient’s deidentified records, along with thousands like them, train AI systems learning to predict treatment outcomes and flag “high-risk” patients.

Dissociative Identity Disorder (DID) remains one of psychiatry’s most misunderstood diagnoses. Defined in the DSM-5-TR as the presence of two or more distinct personality states accompanied by recurrent gaps in memory, DID is characterized by severe disruption of identity and consciousness. Beyond clinical criteria, patients describe losing hours or days, discovering evidence of activities they cannot recall, experiencing themselves as fundamentally fragmented, often associated with profound shame, confusion, and isolation that intensifies when clinicians doubt their reality.

Despite decades of research validating DID’s prevalence, neurobiological correlates, and evidence-based treatment approaches (1, 2), the diagnosis continues to meet clinical skepticism, avoidance, and systematic mislabeling. These responses reveal deeper questions about epistemic authority in psychiatry: whose experiences count as evidence, who gets believed, and how knowledge is constructed and preserved in medical records.

This article examines DID through the lens of epistemic injustice, specifically, Miranda Fricker’s concepts of testimonial injustice (dismissing someone’s account due to prejudice) and hermeneutical injustice (lacking concepts to interpret one’s own experiences). I argue that DID is vulnerable to both forms of epistemic harm, as patients are systematically disbelieved, and mainstream psychiatric frameworks often lack adequate language for understanding dissociative multiplicity.

Critically, these clinical failures are now being encoded into artificial intelligence. Electronic health records train diagnostic algorithms, risk prediction models, and treatment recommendation systems. If these records embed testimonial and hermeneutical injustices – downplaying dissociative symptoms, questioning trauma histories, and pathologizing patients’ attempts to describe their reality, AI will perpetuate and amplify these harms at scale. As AI tools are already being deployed in psychiatric settings, we are encoding decades of epistemic injustice directly into clinical decision-support systems, making these patterns exponentially harder to recognize, challenge, or reverse.

## DID as psychiatry’s epistemic stress test

DID challenges psychiatry to confront whether it genuinely values patient testimony, accommodates complexity, and respects diverse ontological frameworks. The diagnosis resists standard psychiatric models, presenting with multiple, sometimes contradictory identity states within one person that defy neat categorization and provoke clinical unease. This discomfort often manifests as skepticism, and clinicians often suspect malingering, misattribute symptoms to other disorders, or invoke suggestibility. These reactions exemplify testimonial injustice, as we assign credibility deficits based on who the patient is and what they claim to experience.

Psychodynamic theory offers one explanation for this resistance. Fully acknowledging DID requires confronting the reality of extreme, often sadistic trauma, a reality many clinicians find too disturbing to integrate into their clinical schema. The cost of belief, in other words, may feel psychically overwhelming, leading to unconscious defensive skepticism.

Beyond being disbelieved, people with DID face hermeneutical injustice. Most psychiatric training provides insufficient conceptual tools for understanding dissociative symptoms. While DID appears in major diagnostic manuals, trainees receive minimal formal instruction in recognizing dissociation compared to conditions like generalized anxiety disorder, major depressive disorder, or obsessive-compulsive disorder. Validated assessment tools like the Dissociative Experiences Scale (DES) and the Structured Clinical Interview for DSM Dissociative Disorders (SCID-D) exist but rarely appear in core curricula (1). This gap produces under-recognition, misdiagnosis, and overreliance on clinical intuition rather than structured assessment, highlighting the marginalization of complex trauma in psychiatric education. Research demonstrates that these educational deficits directly translate into barriers to care, as patients with dissociative symptoms report difficulty finding knowledgeable providers, face skepticism when they do seek treatment, and often discontinue care due to feeling misunderstood or disbelieved (3).

Without adequate frameworks, both patients and clinicians struggle to make sense of dissociative experiences, resulting in confusion, stigma, and diagnostic error. DID thus serves as a stress test, exposing how fragile psychiatric knowledge becomes when confronted with experiences that do not fit familiar patterns.

## Testimonial and hermeneutical injustice: mechanisms in practice

In clinical settings, testimonial injustice toward DID appears as pervasive doubt, which manifests through questioning patient reports of amnesia or identity shifts, documenting accounts as “inconsistent” or “manipulative,” and treating multiplicity with suspicion rather than clinical curiosity. Hermeneutical injustice emerges when clinicians interpret dissociative symptoms only through deficit-based frameworks, failing to recognize multiplicity as what research demonstrates it to be – a sophisticated adaptive response to overwhelming trauma, a means of preserving some aspect of self when circumstances make psychological wholeness impossible (1, 2). Though often distressing to those who experience it, multiplicity reflects remarkable psychological creativity under unbearable conditions.

These injustices pervade clinical routines: delayed or denied diagnoses, refusal to use patients’ chosen names or pronouns for distinct identity states, treatment plans that actively suppress or ignore dissociative phenomena. The consequences include erasure of patients’ trauma histories and identities. As Grim et al. (4) observe, such practices expose deeper power asymmetries and record-keeping systems that sideline patient perspectives and perpetuate exclusion.

## Documentation as epistemic infrastructure

Clinical documentation actively constructs the narrative that determines how future clinicians will understand a patient. Progress notes, intake summaries, and diagnostic codes are interpretive acts with lasting consequences. When a clinician writes “patient claims multiple identities” or “reports unverified trauma,” the skepticism becomes textual fact, shaping every subsequent clinical encounter.

This is particularly damaging for DID, where capturing complexity is therapeutically essential. Most electronic health record systems cannot adequately represent multiplicity, as they utilize rigid fields for identity, limited space for narrative nuance, dropdown menus that force experiences into predetermined categories. Patients’ accounts become fragmented across disconnected entries, appearing incoherent to readers unfamiliar with dissociative presentations. Over time, this technical artifact is mistaken for the patient’s clinical reality.

Preserving complexity in documentation is clinically necessary. Treatment plans built on oversimplified or skeptical records miss the actual mechanisms maintaining distress, leading to interventions that fail or cause iatrogenic harm. As Hultman & Hultman (5) demonstrate, rigid categorical structures in documentation erode patient agency and compound epistemic harms.

## Clinical consequences: delay, misdiagnosis, alliance rupture

The effects of epistemic injustice are concrete and devastating. Research shows DID diagnosis takes an average of 6 to 12 years (1), during which patients accumulate multiple incorrect diagnoses and undergo treatments that often worsen symptoms. The therapeutic alliance fractures when patients feel disbelieved or dismissed, frequently leading to treatment dropout.

Moreover, these records follow patients to new providers, sometimes through formal chart review, sometimes through the patient’s own recitation of their diagnostic odyssey and failed medication trials, creating a self-reinforcing cycle of skepticism. New clinicians inherit previous biases, approaching patients with preset doubt. This cycle amplifies distress, obstructs recovery, and deepens epistemic harm. As Bergen et al. (6) document, clinical language implying implausibility or inconsistency in patient accounts correlates with worse outcomes and reduced help-seeking. Nester et al. (3) found that individuals with dissociative symptoms identified provider skepticism and lack of specialized knowledge as primary barriers to both accessing treatment initially and continuing care once begun. This finding clearly demonstrates how epistemic injustice compounds across the treatment trajectory.

## From notes to models: how AI learns our injustices

Artificial intelligence systems increasingly use electronic health records to train models for diagnosis, risk stratification, and treatment recommendations. These systems learn whatever patterns exist in their training data, including the assumptions, omissions, and biases embedded there. When records contain testimonial and hermeneutical injustices, skepticism about dissociative symptoms, disbelief of trauma reports, pathologizing language about “inconsistent histories”, models reproduce and scale these harms.

Ferryman et al. (7) argue that biased clinical records are informative artifacts of institutional practices requiring critical examination and correction. Without careful attention to how clinical data are generated, AI risks perpetrating epistemic injustice faster, more widely, and in ways that are increasingly difficult to contest.

This is not a distant concern. AI tools are already being deployed in psychiatric settings. Without intervention, we are encoding decades of epistemic injustice directly into clinical decision-support systems, making these patterns exponentially harder to recognize, challenge, or reverse.

## Interventions and best practices to reduce epistemic injustice

Epistemic injustice in psychiatry involves systematically undervaluing or silencing patient knowledge through testimonial injustice (discrediting patient accounts) and hermeneutical injustice (patients lacking frameworks to interpret their experiences). These harms are especially pronounced in trauma-related conditions like DID, where patient narratives are doubted and poorly integrated into clinical documentation and workflows.

Interventions and best practices to reduce epistemic injustice include:

### Psychiatric training reforms

Integrate validated dissociation screening tools (DES, SCID-D) and trauma measures (PCL-5) into standard intake protocols. Dedicate curriculum time to recognizing dissociative presentations through didactic modules, case conferences with DID-experienced clinicians, and simulation training with standardized patients. Provide explicit supervision on countertransference and defensive reactions to trauma narratives and implement structured decision-making tools with regular diagnostic bias audits, informed by frameworks of epistemic justice and intersectionality (8, 9).

### Soliciting and respecting first-person accounts

Phenomenological approaches encourage clinicians to actively elicit and honor patients’ lived experiences, using structured tools that help patients articulate and understand their stories. This counters testimonial injustice by centering patient voices in documentation and care planning.

### Trauma-informed care principles

Embedding trauma-informed practices (13) such as nonjudgmental language, transparency, patient empowerment, cultural sensitivity, into clinical communication and documentation builds trust and reduces stigma. These principles must be operationalized in workflows, documentation templates, and staff interactions.

### Advocacy and independent mental health advocacy

Advocacy services help patients share experiences and challenge dominant clinical narratives, promoting epistemic justice. While effective for testimonial injustice, advocacy may not fully address hermeneutical injustice due to persistent structural barriers (10).

### Broaching and bridging microskills

In psychotherapy, broaching involves directly acknowledging relevant cultural, social, or systemic factors; bridging creates shared understanding across difference (11). Therapists using these microskills can explicitly discuss power dynamics and work toward epistemic and social justice in cross-cultural therapeutic relationships.

### Legitimizing user knowledge through co-production

Systematically including service user expertise and participatory methods are essential for embedding patient perspectives in mental health systems (4).

### Patient-centered documentation

Using inclusive, strengths-based language and recognizing systemic bias in clinical notes transforms documentation from a site of harm into a therapeutic and advocacy tool, improving patient trust and outcomes (12) (**Table 1**). Allowing patients to access their clinical notes (except in rare circumstances where disclosure poses immediate safety risks) can build trust, correct inaccuracies, and surface epistemic gaps clinicians may not recognize.

**Table 1:** Mechanisms of epistemic injustice in clinical notes and safer alternatives.

Mechanism	Example note language	Clinical/epistemic consequence	Safer alternative phrasing
Testimonial Injustice	“Patient denies involvement in erratic behavior detailed in police report leading to psychiatric assessment, and claims to have no memory of the events.”	Implies disbelief; undermines patient credibility	“Patient reports amnesia to the event.”
Hermeneutical Injustice	“Patient describes herself as having no control over her binge eating. States she is typically highly motivated but ultimately ends up finding herself surrounded by multiple snack wrappers, typically during periods of high stress. Feels sad about this.”	Pathologizes dissociation; may lead to iatrogenic harm from mistargeted psychotropic management, misinterprets trauma signs	“Patient reports fragmented memories and amnesia to binge episodes, suggesting a dissociative component.”
Ontological Doubt	“Patient presented dressed as and claiming to be someone else, consistent with delusions documented during prior hospitalization. This is concerning for psychotic decompensation.”	Erases subjective reality; delays diagnosis, flattens dissociative switching into psychosis framework; may lead to inappropriate antipsychotic treatment	“Patient presented using different name and reported being age 7. Denies awareness of adult identity in this state. No formal thought disorder or perceptual disturbances noted. Consider identity state switching; recommend dissociative assessment”
Documentation Flattening	“Patient denies traumatic events. No history of trauma.”	Neglects dissociative mechanisms; flattens trauma into single-event encounters, does not assess for complex trauma features.	“ Patient denies single-event life-threatening trauma but describes relational trauma including profound childhood neglect and navigation of the foster care system. Assess for complex trauma presentation.”
Misattribution	“Patient uncooperative with history taking, adding interactive complexity by perseverating on concluded aspects of the interview. Behavior consistent with known borderline features (help- seeking, help-rejecting.) “	Stigmatizes; diverts from accurate diagnosis	“Although they denied dissociative episodes, patient could not recall the first 15 minutes of session. Denied awareness of time loss when asked.”

### Implementation strategies

Creating lived-experience roles with appropriate support and compensation, as well as training staff in trauma-informed care and testimonial awareness. Involving patients and communities in documentation design, education, and service planning.

These interventions face real obstacles, such as limited resources in training programs, institutional resistance to changing established documentation workflows, and the risk that even well-intentioned inclusion of patient voices becomes tokenistic if not accompanied by genuine power-sharing. Implementation requires sustained institutional commitment and resource allocation. As such, advocacy for this population represents a meaningful step for every clinician to take towards systemic justice.

## Governance reforms: dataset statements, inclusion audits, redress mechanisms

AI governance must treat clinical data as ethically consequential infrastructure. Dataset statements should explicitly document how data were generated, which perspectives were excluded, and what assumptions shaped documentation practices. Inclusion audits can assess whether marginalized diagnoses like DID are adequately represented and accurately characterized in training datasets.

Finally, redress mechanisms are essential. Patients must have pathways to challenge inaccuracies in their records and meaningful input into how their data are used in research and algorithm development. These safeguards help ensure AI systems advance justice rather than amplify harm (14).

## Conclusion: centering epistemic equity for complex trauma

DID exposes fundamental weaknesses in psychiatry’s epistemic practices and the dangers of encoding those weaknesses into AI systems. Patients with DID demand that the field listen more carefully, document differently, and reconsider what constitutes credible knowledge. By treating clinical narratives as valid sources of knowledge and reforming how they are generated and utilized, psychiatry can move toward greater epistemic justice.

This work matters urgently. We are establishing the foundation for ethical and equitable deployment of AI in mental health care delivery. Importantly, this work also improves care for some of psychiatry’s most vulnerable patients. The choices we make now about clinical documentation, training, and data governance will shape algorithmic psychiatry for decades. DID patients have waited long enough to be believed. We cannot afford to scale their suffering into our technological future.

## Data Availability

The original contributions presented in the study are included in the article/supplementary material. Further inquiries can be directed to the corresponding author/s.
